# The Gail Model and Its Use in Preventive Screening: A Comparison of the Corbelli Study

**DOI:** 10.7759/cureus.56290

**Published:** 2024-03-16

**Authors:** William R Pruitt, Beryl Samuels, Scott Cunningham

**Affiliations:** 1 Preventive Medicine, Oceania University of Medicine, Apia, WSM; 2 Neurosciences, Johns Hopkins University, Baltimore, USA; 3 Obstetrics and Gynecology, All American Institute of Medical Sciences, Black River, JAM

**Keywords:** gail score, bcrat, risk assessment tool, preventive screening, risk calculator, chemoprevention, gail model, breast cancer

## Abstract

Background

This study aims to determine the usage of the Gail model in screening for breast cancer during physical examinations of women by sampling primary care physicians in two regions of Texas - Hidalgo County and Johnson County. A Gail score of 1.66% or higher indicates increased breast cancer risk. Three specialties are surveyed: internal medicine (IM), family medicine (FM), and gynecology (GYN). The null hypothesis for this study is that primary care physicians do not use the Gail model in screening for breast cancer during physical examinations of women.

Methods

A survey was distributed to 100 physicians with specialties in IM, FM, and GYN from May 2022 to July 2022. The survey assessed the physician’s frequency of use of the Gail model and chemoprevention. Data were collected by distributing survey questionnaires to physicians in person. Descriptive statistics were used for response distributions. Fisher's exact probability test was used for comparisons across specialties.

Results

The response rate was 34% (34/100). Thirty-eight percent of the physicians surveyed reported using the Gail model in their practice (IM 46%, FM 23%, and GYN 31%). All 13 of the physicians using the Gail model were open to using chemoprevention.

Conclusions

Only 38% of the physicians surveyed responded that they use the Gail model in their practice. The study concluded that a minority of primary care physicians used the Gail model to decrease breast cancer risk. Further research would help to define better the Gail model and its use in preventing breast cancer in women. The Gail model appears to be beneficial to breast cancer risk reduction; however, risk reduction medication side effects need to be minimized.

## Introduction

The incidence of breast cancer is predicted to continue rising over the next 20 years [1]. Early age of menarche, late age of first pregnancy, fewer pregnancies, decreased breastfeeding, and later onset of menopause are all cited as major breast cancer risk factors. Other risk factors include obesity, alcohol consumption, lack of exercise, and hormone replacement therapy (HRT) [1]. Non-modifiable risk factors include female gender, older age, family history, genetic mutations, race/ethnicity, and estrogen exposure. Modifiable risk factors include hormonal replacement therapy, diethylstilbestrol, physical activity, overweight/obesity, alcohol intake, smoking, vitamin deficiency, excessive exposure to artificial light, intake of processed food, exposure to chemicals, and other drugs [2]. In the United States, the American Cancer Society (ACS) estimates new cases in 2023 to be 297,790 and 2,800 for women and men, respectively [3]. Estimated deaths from breast cancer in 2023 are 43,170 and 530 for women and men, respectively. According to the ACS in 2022, there were 51,400 ductal carcinoma-in-situ (DCIS) cases, 287,850 cases of invasive breast cancer, and 43,250 deaths [4]. In 2020, the Centers for Disease Control and Prevention listed the mortality from breast cancer as second only to lung and bronchus cancer in the United States [5]. According to Corbelli et al. (2014), there has been less focus on breast cancer risk reduction and risk reduction medications [6].

The Gail model (also known as the Breast Cancer Risk Assessment Tool (BCRAT)) was developed by Mitchell H. Gail in 1989 [7]. It predicts both the five-year and lifetime risk of invasive breast cancer. Women with a score of 1.66% or higher are considered to be at increased risk for breast cancer [8].

The Gail model asks a series of questions, which include a medical history of breast cancer, a mutation in the BRCA1 or BRCA2 gene, age, age at first menstrual period, age at first live birth, first-degree relatives with breast cancer, breast biopsy, and race/ethnicity. 

The Gail risk score calculator is not recommended for women with a BRCA1 or BRCA1 mutation, previous history of lobular carcinoma in situ (LCIS) or ductal carcinoma in situ (DCIS), women with Li-Fraumeni mutation, or those who have had radiation therapy to the chest [8,9]. Referral to a medical geneticist is recommended for Li-Fraumeni syndrome and other inherited causes [8].

The International Breast Cancer Intervention Study (IBIS) Breast Cancer Risk Evaluation Tool is used with women who have had a previous LCIS. This tool determines the risk of DCIS or invasive breast cancer (http://www.ems-trials.org/riskevaluator/) [8,10].

The Breast and Ovarian Analysis of Disease Incidence and Carrier Estimation Algorithm (BOADICEA) (http://ccge.medschl.cam.ac.uk/boadicea/) is used for calculating risks for women with a history of breast and ovarian cancer. It is also used for determining the risk of being a carrier of the BRCA1 and BRCA2 gene mutations [8,11].

Sa-Nguanraksa et al. (2019) showed that the Gail model underestimated breast cancer risk in Thai women [12]. A possible reason may be due to environmental factors and lifestyle having a greater effect than exposure to estrogen. Atypical ductal hyperplasia (ADH) was the strongest risk factor [12]. Nickson (2018) investigated 40,158 women ages 50-69 in Australia and determined that the Gail model was effective at determining invasive breast cancer risk for five years after assessment [13]. Abdel-Razeq et al. (2020) studied women in Jordan. Family history was the primary factor in determining breast cancer risk. The use of the Gail model was shown to detect only 17.3% of women if the score was determined five years before a diagnosis [14]. This shows a limitation of the Gail score in that it was developed to measure breast cancer risk in a population of American women. Mackarem et al. (2001) found that the Gail model did not identify the immediate risk of breast cancer in women under the age of 40 [15]. Bener et al. (2017) studied the performance of the Gail model in Qatari women [16]. The Gail model was found to be appropriate for measuring breast cancer risk in Qatari women [16].

Corbelli et al. (2014) was the first published study that the author discovered that compared the use of the Gail model in different medical specialties [6]. This study consisted of a web-based survey of resident and attending physicians at the University of Pittsburgh Medical Center. Three specialties were included in the survey: internal medicine (IM), family medicine (FM), and gynecology (GYN). These physicians were required to be university or community-based doctors who practiced at least one half-day per week in an outpatient setting. The Corbelli survey occurred in April 2012. Corbelli et al. (2014) concluded that a minority of primary care physicians used the Gail model (BCRAT) to prevent or to decrease breast cancer risk [6]. The present study aims to determine if there are any significant differences from the Corbelli study (2014) [6] in the use of the Gail model and breast cancer preventive therapy with a sample of non-university-based primary care physicians in Hidalgo County and Johnson County, Texas. The survey and data gathering occurred during the Spring 2022 and Summer 2022.

The U.S. Food and Drug Administration has approved tamoxifen and raloxifene for use in breast cancer prevention. These medications are also known as selective estrogen receptor modulators (SERMs) [17]. 

Tamoxifen (Nolvadex) binds to estrogen receptors and competitively blocks the binding of endogenous estrogens. In breast tissue, tamoxifen acts as an estrogen receptor antagonist. It inhibits breast cancer cell proliferation in patients who are estrogen receptor-positive. Side effects of tamoxifen include nausea, vomiting, hot flashes, vaginal bleeding, and menstrual irregularities. It increases the risk of endometrial cancer due to the proliferation of endometrial cells. Raloxifene (Evista) is also an SERM that is an antagonist in the breast and uterus and a partial agonist in bone. It helps reduce the risk of breast cancer in postmenopausal women. Raloxifene is not associated with an increased risk of endometrial cancer [18]. 

Both tamoxifen and raloxifene have been shown to significantly reduce breast cancer risk. The use of SERMs is recommended by the United States Preventive Services Task Force (USPSTF) for women who have an increased risk for breast cancer and a low side-effect profile. However, the USPSTF has recommended against routine use of tamoxifen and raloxifene at low or average risk for breast cancer [19]. The American Society of Clinical Oncology recommends the use of tamoxifen or raloxifene as an option for treating ER-positive breast cancer in premenopausal women who are 35 years and older with a five-year Gail score of greater than or equal to 1.66%. Tamoxifen or raloxifene is recommended for women with LCIS. Contraindications for these medications include deep vein thrombosis (DVT), pulmonary embolus (PE), stroke, transient ischemic attack (TIA), or patient immobilization. Pregnant women, those who may become pregnant, and nursing mothers should avoid tamoxifen. Moreover, tamoxifen is not recommended while using hormone medications [20].

Khaliq et al. (2016) studied the use of chemoprevention in hospitalized women at high risk for breast cancer. The study was performed at Johns Hopkins Bayview Medical Center and found there were not any women identified as high risk who were using chemoprevention for breast cancer risk reduction [21].

The Breast Cancer Prevention Trial (BCPT) showed that women with a history of atypical hyperplasia (AH) benefited more from preventive therapy. This trial showed an 86% risk reduction in women with AH. BCPT data also showed that women under 50 years were less likely to be harmed by the preventive therapy [22].

The study by Tchou et al. (2004) found that only atypical hyperplasia and hysterectomy were significant risk factors. This study concluded that the risk due to AH or LCIS was the primary risk factor influencing tamoxifen treatment's offering and acceptance [23].

## Materials and methods

Data source and study survey

The Institutional Review Board (IRB) committee at the Oceania University of Medicine approved this study (IRB number: 21-0914WP). Physicians in three primary care specialties, namely, IM, FM, and GYN, were surveyed by using a written questionnaire (refer to Appendix). The objectives of this study are to determine the use and knowledge of the Gail model by primary care physicians in two areas of Texas. The findings of the present study will be compared with a previous study by Corbelli et al. [6].

The sample population consisted of private practice physicians in Johnson County, Texas, and Hidalgo County, Texas. Physicians in the sample were located using the Google search engine. Search terms consisted of "internal medicine," "family medicine," "gynecology," "physicians," "Hidalgo County, Texas," and "Johnson County, Texas." The sample size of 100 was selected from physicians located in proximity to the primary author's residence. There were more physicians in Hidalgo County than in Johnson County. This led to taking the sample of 85 in Hidalgo County and 15 in Johnson County. The sample was selected based on proximity to the primary author's residence and physician identification in one of the three specialties of IM, FM, and GYN. The sample was taken from private practice physicians only.

The survey was performed during the spring/summer of 2022. The two Texas locales were selected for convenience. The primary author resides in both locations. The questionnaires were distributed by the primary and secondary authors visiting the physician clinics and offices. Informed consent from the physicians was not obtained, but the study was approved by the Oceania University of Medicine IRB, which was explained in the survey cover letter. A self-addressed stamped envelope and cover letter were attached to each questionnaire. The questionnaire was anonymous with no identifying information. Completion of the questionnaire required about five minutes. Survey questions were based on the Corbelli study (2014) [6].

The survey questionnaire consisted of 10 questions. The first three survey questions were demographic: medical specialty, experience, and gender. The fourth question asked if the physician used the Gail model (yes or no). If no, the respondent skipped to question 10. If yes, the survey continued with questions 5 through 9. Question 5 asked about the application of the Gail score. Questions 6 through 10 concerned the use of chemoprevention.

Inclusion criteria

Inclusion criteria consisted of private practice physicians in three medical specialties (IM, FM, and GYN) located in the Texas counties of Johnson and Hidalgo. Other Texas counties and medical specialties were not surveyed. The Google search engine was used to identify each physician's medical specialty credentials and private practice location.

Statistical analysis

Due to the small sample size, Fisher's exact probability was calculated using Vassarstats: statistical computation web site [24]. The Freeman-Halton extension of the Fisher exact probability test for two rows and three columns was utilized. Data from the completed and returned questionnaires were inputted into the VassarStats online calculator 2 x 3 table to compute Fisher's exact probability. Variable 1 is the use of the Gail model and chemoprevention. Variable 2 is the physician's medical specialty (IM, FM, and GYN).

## Results

Of the 100 questionnaires distributed, there were 34 responses. The physicians responding consisted of three specialties: 38% IM, 47% FM, and 15% GYN. Twenty-three percent had five years or less experience as a physician. Thirty-two percent of the physicians who responded had greater than 20 years of experience as a physician. Sixty-two percent of the respondents were male physicians, and 38% were female physicians (refer to Table 1). 

**Table 1:** Characteristics of the survey participants

Characteristic	Responses	%
Medical specialty:		
Internal medicine	13	38%
Family medicine	16	47%
Gynecology	5	15%
Total (n)	34	100%
Years of experience as a physician:		
<5 years	8	23%
6-10 years	4	12%
11-15 years	5	15%
16-20 years	6	18%
>20 years	11	32%
Total (n)	34	100%
Gender:		
Male	21	62%
Female	13	38%
Total (n)	34	100%

Thirty-eight percent of the responding physicians reported the use of the Gail score in their practice (46% IM, 23% FM, GYN 31%) (refer to Table 2). A majority of physicians responded that they would use the Gail model in women who may be at a higher-than-average risk for breast cancer. Fifty-four percent stated that they would use the Gail score in women who may be at a higher-than-average risk for breast cancer. Forty-three percent answered that they were not sufficiently familiar with the Gail score. Nineteen percent answered that they did not think the results of the Gail score would change their management (refer to Table 2).

**Table 2:** Use of the Gail model and barriers to use Fisher's exact probability: P(a) = 0.032. Variable 1: use of the Gail model; variable 2: medical specialty.

Variable	Responses	%
Use of the Gail model:		
Yes	13	38%
No	21	62%
Total (n)	34	100%
Proportion using Gail by specialty:		
Internal medicine	6	46%
Family medicine	3	23%
Gynecology	4	31%
Total (n)	13	100%
Usage of the Gail was applied in the following situations:		
Women over 35 years of age	2	15%
Women over 60 years of age	0	0%
Women with a history of breast cancer	4	31%
Women who may be at a higher-than-average risk for breast cancer	7	54%
Total (n)	13	100%
Reasons for not using the Gail model:		
"I do not see patients in whom calculation of the Gail score is indicated."	7	33%
"I do not have enough time with my patients to use the Gail score."	1	5%
"I do not think that the results of the Gail score would change my management."	4	19%
"I am not sufficiently familiar with the Gail score."	9	43%
Total (n)	21	100%

Of the physicians using the Gail model in their practice, all responded that they use chemoprevention (refer to Table 3). It appears that many of the physicians did not have correct knowledge of the Gail score percent needed to consider the use of chemotherapy. The Gail model recommends a score of 1.66% or higher for considering the use of chemoprevention. Eighty percent of the physicians answered that they had not identified a patient in whom chemoprevention was indicated.

**Table 3:** Chemoprevention and barriers to use Fisher's exact probability: P(a) = 1.0. Variable 1: use of chemoprevention; variable 2: medical specialty.

Variable	Responses	%
Use of chemoprevention:		
Yes	13	100%
No	0	0%
Total (n)	13	100%
The proportion using chemoprevention by specialty:		
Internal medicine	6	46%
Family medicine	3	23%
Gynecology	4	31%
Total (n)	13	100%
Among providers using chemoprevention, the minimum Gail score required to recommend:		
1-2%	2	15%
3-5%	9	70%
6-10%	2	15%
11-15%	0	0%
>15%	0	0%
Total (n)	13	100%
Providers using chemoprevention, number of times prescribed:		
None	2	15%
1 to 5	4	31%
6 to 10	2	15%
11 to 20	2	15%
>20	3	24%
Total (n)	13	100%
Reasons for not using chemoprevention: (only 10 of the 21 physicians not using the Gail Model responded)		
"I have not identified a patient in whom chemoprevention was indicated."	8	80%
"I do not believe that chemoprevention benefits most women who are eligible to receive it."	0	0%
"I am not comfortable prescribing chemoprevention."	2	20%
"I do not have time to discuss chemoprevention with my patients."	0	0%
Total (n)	10	100%

## Discussion

The current study compares the findings of the study by Corbelli et al. (2014). The two studies produced similar conclusions. Corbelli et al. found that only 41% of the physicians reported the use of the Gail model. This result is very similar to the present study with 38% reporting the use of the Gail model. In the Corbelli study, 54% of the responding physicians were IM, 24% FM, and 21% GYN versus 38% IM, 47% FM, and 15% GYN in the current study. Physicians who responded were 47% male and 53% female versus 62% male and 38% female in the current study (refer to Table 1). Corbelli et al. showed that 59% of the physicians stated that they did not use the Gail model versus 62% in the current study {refer to Table 2) [6]. Providing more information to physicians about the Gail model would likely help increase its use. The National Institutes of Health: National Cancer Institute website provides a Gail score calculator (https://bcrisktool.cancer.gov/calculator.html) [8].

In comparison, the Corbelli (2014) study sampled resident and attending physicians at the University of Pittsburgh Medical Center who were university- or community-based. The category of residents (first year through fourth year) or attending physicians was identified. These physicians were practicing at least one half-day per week in an outpatient office or clinic. The Corbelli study participants accessed a public survey URL that was sent as an email. Consent was obtained through e-mail. A random drawing was offered to win one of two iPad computers. The Corbelli study included 575 physicians, of which 316 participated. The survey occurred from February to April 2012 [6].

Both the Corbelli study (2014) and the present study showed that there was low use of the Gail model. One barrier to use described by Brewster et al. (2005) found that a physician would need to screen five or six women aged 40 to 70 years to find one woman eligible to be counseled for breast cancer chemoprevention. The study participants were members of the CLUE cohort, which is based in Washington County, Maryland. The study found that an estimated 18% of white women aged 40-70 would meet the criteria for chemoprevention counseling. Brewster found that 26 women aged 40-49 years and 142 women from ages 60 to 70 years with a uterus would need to be screened with the Gail model or another model to find one woman eligible to receive counseling on chemoprevention and to have a benefit-risk index for use the of tamoxifen. Brewster et al. suggested considering the differences between clinical trial populations and community populations under study. Brewster et al. found that women in clinical trials are generally healthier than women in other patient populations [25]. Salant et al. (2006) found that many women did not consider themselves high risk for breast cancer because they did not have any signs or symptoms [26]. A five-year Gail score of 1.67 or higher was used to meet the clinical threshold for the use of SERMs. Salant et al. (2006) noted that breast cancer is not often put at the top of the list of health concerns. Many women consider breast cancer risk to fluctuate. The high risk is considered depending on physical signs and symptoms. Most of the women in the Salant study thought that cancer was started by stress or worry. Women were not interested in taking medications for breast cancer [26]. In the current study, there were only 38% of physicians who used the Gail score (refer to Table 2). Salant et al. (2006) suggested that a possible explanation for the low use of the Gail score might be that women did not relate to a numerical score [26]. The low survey response rate might have been due to a lack of interest or knowledge of the Gail score model, breast cancer screening, and risk reduction medications. It is important to improve the use of the Gail model to help prevent and lower the risk of women developing breast cancer, which is currently the number one cancer in women in terms of estimated new cases in the United States [3].

Improving the use of the Gail model may help to improve the use of chemoprevention. The USPSTF recommends that women at increased risk for breast cancer who have a low risk for side effects should be offered risk-reducing medications, such as tamoxifen or raloxifene. The USPSTF found that treatment with tamoxifen or raloxifene can significantly reduce the relative risk for ER-positive breast cancer in post-menopausal women [27]. In the present study, 34% of the physicians who did not use the Gail model stated that they did not see patients in whom the Gail score calculation is indicated versus 17% in the Corbelli study (2014). Nineteen percent of the physicians responded that the Gail score would not change their management versus 26% in the Corbelli study (2014). Forty-three percent of the physicians not using the Gail model responded that they were not familiar with the Gail score (refer to Table 2). Corbelli et al. (2014) found that 82% were not sufficiently familiar with the Gail score vs. 43% in the current study [6]. The small sample size of the current study may account for some of the differences. 

The main strength of the Gail model is that it can inform women of their breast cancer risk. Other studies of different populations have associated age, age of first birth, age at menarche, family history, menopausal status, and parity with breast cancer risk [28]. The weakness of the Gail model is that it leaves out some risk factors when applied to different ethnicities and populations. Different populations have shown different results in its use. Iranian women had a lower discriminatory power in determining breast cancer risk [29]. The breast cancer risk in women with atypical hyperplasia of the breast may be underestimated with the Gail model. Risk assessment could be improved by using tissue-based risk factors with a biopsy [30].

The limitations of the current study include the small sample size. One hundred questionnaires were distributed to physicians, but only 34 participated. The current study did not survey other methods of breast cancer screening. The patient profiles of the clinics were not available to the authors.

## Conclusions

The data collected in this study show that primary care physicians in the specialties of IM, FM, and GYN who practice in Hidalgo County, Texas, and Johnson County, Texas, do not frequently utilize the Gail model to identify and estimate the risk of breast cancer in women. Further research would help to define better the Gail model and its use in preventing breast cancer in women. The Gail model appears to be beneficial to breast cancer risk reduction; however, risk reduction medication side effects need to be minimized.

## Appendices

Survey Questionnaire modified from the Corbelli study (2014):

1.     What is your specialty?

Internal medicine

Family medicine

Gynecology

2.     Years of experience as a physician?

Less than 5 years

6-10 years

11-15 years

16-20 years

Greater than 20 years

3.     Gender?

Male

Female

4.     Do you use the Gail Model in your practice? 

Yes

No

If no, please skip to question 10.

5.     If yes, which of the following situations would apply to a Gail score use:

Women over 35 years of age

Women over 60 years of age

Women with a history of breast cancer

Women who may be at higher-than-average risk for breast cancer.

6.     Do you prescribe medications for breast cancer prevention? 

Yes

No

7.     What is the minimum Gail score required to recommend chemoprevention?

1-2%

3-5%

6-10%

11-15%

Greater than 15%

8.     How many times have you prescribed chemoprevention?

0

1-5

6-10

11-20

Greater than 20

9.     If not using chemoprevention, which choice below would you most agree with?

I have not identified a patient in whom chemoprevention was indicated.

I do not believe that chemoprevention benefits most women who are eligible to receive it.

I am not comfortable prescribing chemoprevention.

I do not have time to discuss chemoprevention with my patients.

10.   If not using the Gail model, which choice below would you most agree with?

I do not see patients in whom the calculation of the Gail score is indicated.

I do not have enough time with my patients to use the Gail score.

I do not think the results of the Gail score would change my management.

I am not sufficiently familiar with the Gail score.

Please write your address if you would like a copy of the study results.

E-mail address: ____________________________________________
